# Bioinspired cardiac-targeted metal-organic framework nanozyme for modulating inflammatory responses in heart failure with preserved ejection fraction

**DOI:** 10.3389/fbioe.2026.1744643

**Published:** 2026-02-12

**Authors:** Yuesheng Gui, Xiaowan Fan, Kairui Xiao, Junyue Xing, Zongfeng Niu, Yingying Wang, Weining Yuan, Jia Shen, Yingchao Shi, Xiaolei Cheng, Yu Han, Zhen Li, Hao Tang

**Affiliations:** 1 Zhengzhou University People’s Hospital, Henan Provincial People’s Hospital, Zhengzhou, Henan, China; 2 Zhengzhou Key Laboratory of Cardiovascular Aging, Henan Key Laboratory of Chronic Disease Management, Henan Province Key Laboratory for Prevention and Treatment of Coronary Heart Disease, National Health Commission Key Laboratory of Cardiovascular Regenerative Medicine, Central China Subcenter of National Center for Cardiovascular Hospital and Fuwai Central China Cardiovascular Hospital, Zhengzhou, Henan, China; 3 School of Medicine of Henan University, Zhengzhou, Henan, China; 4 Institute of Cardiovascular Disease, Henan Academy of Innovations in Medical Science, Zhengzhou, Henan, China

**Keywords:** cardiac-targeted, HFpEF, Iinsulin resistance, nanozyme, ROS

## Abstract

**Introduction:**

Heart failure with preserved ejection fraction (HFpEF) is a common heart failure type with poor prognosis. Its mechanisms are unclear, and specific diagnostic criteria and effective treatments are lacking. Recent studies have emphasized the impact of inflammation and oxidative stress on the occurrence and development of HFpEF. Anti-inflammatory interventions targeting oxidative stress show promise, but traditional antioxidants are insufficient.

**Methods:**

A biomimetic manganese‐doped ZIF‐8 nanozyme (MnZIF) was synthesized. It was further modified with atrial natriuretic peptide (ANP) to create a cardiac‐targeted nanozyme, NanoAM. Its efficacy was evaluated in a murine HFpEF model induced by a high‐fat diet and L‐NAME. Assessments included echocardiography, pressure-volume loop analysis, histology, and transcriptomics. *In vitro* studies measured reactive oxygen species (ROS) scavenging, cytotoxicity, and glucose uptake mechanisms.

**Results:**

NanoAM exhibited multi‐enzyme mimetic (SOD/CAT) activity and demonstrated excellent cardiac targeting and biocompatibility *in vivo*. In HFpEF mice, NanoAM significantly alleviated diastolic dysfunction, lowered blood pressure, and reduced cardiac fibrosis and hypertrophy. Mechanistically, NanoAM effectively scavenged myocardial ROS and downregulated pro‐inflammatory cytokines. Transcriptomic and biochemical analyses revealed that NanoAM suppressed the expression of SOCS3, leading to enhanced IRS1‐AKT2 signaling and increased GLUT4 membrane translocation.

**Discussion:**

This study develops a novel cardiac-targeted nanozyme that effectively ameliorates key pathologies in experimental HFpEF. Its therapeutic action involves a dual mechanism: direct ROS scavenging and modulation of the SOCS3‐IRS1‐AKT2 signaling axis to improve insulin resistance. These findings highlight the potential of multifunctional nanozymes as a promising strategy for tackling the complex pathophysiology of HFpEF.

## Graphical Abstract

**Figure:**
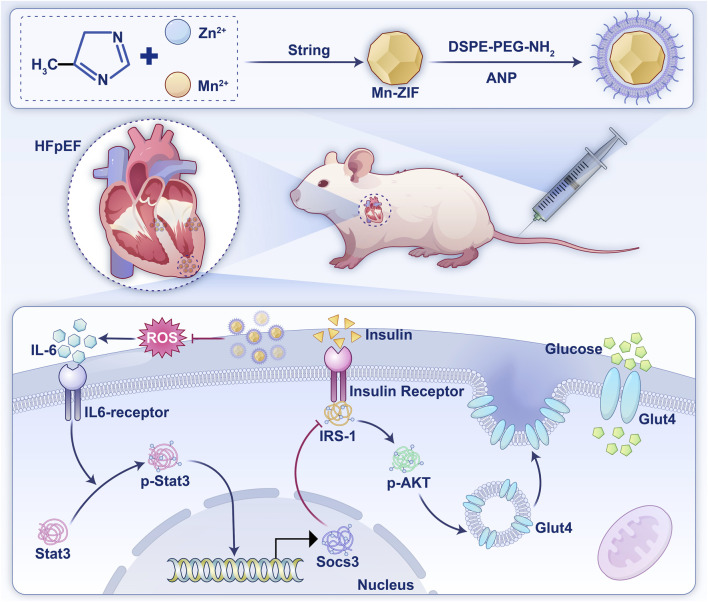

## Introduction

Heart failure with preserved ejection fraction (HFpEF) has become the predominant subtype of heart failure, accounting for over 50% of all heart failure cases (Abdin et al., 2024). Patients with HFPEF present with typical heart failure symptoms (i.e., dyspnea, reduced exercise capacity), while having a normal left ventricular ejection fraction (>50%) (Mishra and Kass, 2021). Although significant progress has been made in the pharmacological treatment strategies for heart failure with reduced ejection fraction (HFrEF) over the past few decades, drug therapies for HFpEF remain highly limited, which may be partly attributed to the complexity and high heterogeneity of its pathogenesis (Zakeri and Cowie, 2018). Clinically, patients with HFpEF often present with metabolic syndrome characterized by endothelial dysfunction and systemic inflammation, including conditions such as obesity, type 2 diabetes, and hypertension (Bode et al., 2020). The resulting systemic inflammation stimulates cardiac endothelial cells to produce reactive oxygen species (ROS), adversely affecting myocardial remodeling and both systolic and diastolic cardiac function (Franssen et al., 2016). Therefore, these comorbidities may play a critical pathophysiological role in promoting myocardial stiffness, fibrosis, and diastolic dysfunction in HFpEF patients through mechanisms involving systemic microvascular inflammation and coronary microvascular dysfunction.

Increased evidence suggests that excessive production of reactive oxygen species (ROS) and oxidative stress appear to play a pivotal role in the pathogenesis of HFpEF (Martinez et al., 2024; Schiattarella et al., 2019). Against the backdrop of low-grade systemic inflammation, myocardial microvascular endothelial inflammation induces the production of massive amounts of reactive oxygen species (ROS), creating a vicious cycle of oxidative and nitrosative stress (Staiculescu et al., 2014). ROS directly impair the NO-cGMP-PKG signaling pathway, leading to decreased cyclic guanosine monophosphate (cGMP) levels and suppressed PKG activity, which in turn causes disrupted calcium regulation and diastolic dysfunction in cardiomyocytes (Cai et al., 2023). Simultaneously, excessive accumulation of mitochondrial ROS further disrupts mitochondrial membrane potential and energy metabolism, exacerbating myocardial hypertrophy and fibrosis (Suski et al., 2011). These mechanisms collectively constitute the oxidative stress framework underlying the pathogenesis of HFpEF, suggesting that scavenging ROS and restoring antioxidant defenses (e.g., activating the Nrf2-HO-1 pathway) may reverse myocardial structural remodeling and improve clinical prognosis (Loboda et al., 2016).

Nanozymes are a class of nanomaterials with enzyme-like activity that can mimic the activity of antioxidant enzymes such as superoxide dismutase (SOD) and catalase (CAT) (Xu et al., 2022). They possess the advantages of high stability, tunable size, and multifunctional surface modification (Wu et al., 2025). In recent years, nanozymes have been systematically evaluated for oxidative stress intervention in cardiovascular diseases (Zou et al., 2025). Studies have demonstrated their ability to efficiently scavenge excess ROS *in vivo*, improve endothelial function, and inhibit myocardial fibrosis (Gu et al., 2024). In animal models of heart failure with end-stage renal failure (HFpEF), nanozymes targeting mitochondrial ROS (such as Pt@MitoLipo) significantly reduced myocardial diastolic blood pressure and improved left ventricular compliance by precisely targeting and eliminating mitochondrial superoxide, demonstrating potential therapeutic value for metabolically driven HFpEF (Xue et al., 2022). The multi-enzyme cascade properties of nanozymes (exhibiting simultaneous SOD, CAT, and GPX activities) enable them to simultaneously suppress oxidative stress and inflammatory signaling in metabolically disordered environments, potentially offering a novel therapeutic strategy for related myocardial metabolic disorders (Wang et al., 2024). Nanozymes, with their powerful antioxidant function and designable targeting properties, are gradually becoming a cutting-edge research direction for intervening in HFpEF and other cardiovascular diseases, laying a scientific foundation for subsequent nanozyme-mediated intervention strategies.

In this study, we developed a cardiac-targeted nanozyme NanoAM modified with atrial natriuretic peptide (ANP), which ameliorates cardiac diastolic dysfunction by scavenging ROS and simultaneously modulating insulin resistance pathway, thereby reducing blood pressure, attenuating cardiac fibrosis and hypertrophy, and ultimately treating HFpEF in mice.

## Materials and methods

### Study approval

This study was approved by the Ethics Committee of the Central China Branch of the National Center for Cardiovascular Diseases (approval number: FZX-IACUC-2024005). All animal manipulations were performed in accordance with procedures reviewed and approved by the Laboratory Animal Care Committee. Every effort was made to minimize animal suffering.

### Cell counting Kit-8 (CCK8) assay

AC16 cells were cultured to reach 70%–80% confluence and treated with NanoAM at various concentrations (0, 3.125, 6.25, 12.5, 25, 75, 100, 150, 180, 200, and 300 μg/mL) for 24 h, 10 µL of CCK-8 (APExBIO, K1018) was added to each well for an additional hour. Absorbance was measured at 450 nm using a microplate reader (Full wavelength microplate reader, thermo).

### Flow cytometry

Flow cytometry was utilized to evaluate the reactive oxygen species (ROS) levels in AC16 cells. The cells were divided into three groups: control group, H_2_O_2_-treated group, and H_2_O_2_+NanoAM-treated group. After pretreatment with NanoAM (15 mg/mL) for 6 h, H_2_O_2_ (300 nM) was added to induce for 24 h, we counted the fluorescence intensity of FITC.

### Dichloro-dihydro-fluorescein diacetate (DCFH-DA) assay

AC16 cells were pretreated with 15 mg/kg NanoAM for 6 h and then induced with H_2_O_2_ (300 nM) for 24 h.10 μM DCFH-DA working solution (Beyotime, S0035S) was added and incubated at 37 °C for 30 min. Intracellular ROS levels were assessed by observing green fluorescence using a fluorescence microscope (Nikon, SAP18, excitation wavelength 485 nm, emission wavelength 530 nm)

### JC-1 assay for mitochondrial membrane potential

AC16 cells were cultured and treated with NanoAM or H_2_O_2_ as described before. 10 μM JC-1 working solution (Biotime, C2006) diluted in serum-free DMEM) was added for 15 min. Green fluorescence (JC-1 monomers, indicating decreased mitochondrial membrane potential) and red fluorescence (JC-1 aggregates, indicating normal mitochondrial membrane potential) were observed using a fluorescence microscope (excitation wavelength 485 nm, emission wavelength 530 nm; excitation wavelength 585 nm, emission wavelength 590 nm). Changes in mitochondrial membrane potential were assessed by calculating the ratio of greeen to red fluorescence.

### Mouse model

Six-weeks-old C57BL/6N mice were purchased from GemPharmatech (Jiangsu, China) and randomly divided into 3 groups: NC, HF(HFpEF), and HFi(HFpEF + NanoAM), (n = 6 per group), Mice in HF group was fed a high-fat diet (ResearchDiets, D12492, 60 kcal% fat) and 0.5 g/L L-NAME (N(ω)-nitro-L-arginine methyl ester hydrochloride) in drinking water for 12 weeks, while mice in NC group were fed with normal diet (Schiattarella et al., 2019). The mice in HFi group were given NanoAM (15 mg/kg) by tail vein injection twice a week based on the dietary strategy of the HF group. Then the mice underwent ultrasound examination (Fujifilm VisualSonics), blood pressure measurement (KENT Scientific, CODA4) and pressure-volume detection (AD Instruments, PL2604).

### Living image

The targeting efficiency of NanoAM was evaluated using a small animal *in vivo* imaging system (IVIS Spectrum, United States). Briefly, C57BL/6N mice with an average weight of about 30 g were divided into a Cy5.5-NanoAM experimental group, a Cy5.5-NanoM non-targeted control group, and a solvent group. The dose was 15 mg/kg injected into the tail vein and the mice were tested 6 h later.

### Blood pressure measurement

Mouse blood pressure was measured using a high-precision tail artery sphygmomanometer (KENT Scientific, CODA4). During the experiment, mice were placed on a temperature-controlled heating plate, covered with a black blanket to minimize stress caused by light stimulation, and then gently placed in a mouse restrainer to prevent injury. The sphygmomanometer, precisely attached to the mouse’s tail, monitored blood pressure changes in real time and calculated key parameters such as systolic, diastolic, and mean arterial pressure.

### Echocardiography

When performing cardiac ultrasound examination of mice, it was used to induce and maintain mice. The mice were anesthetized with isoflurane (2% concentration) and fixed on a heating platform in a supine position to maintain the body temperature, and the chest hair was removed with depilatory cream to facilitate the contact of the ultrasound probe. Echocardiography was performed by vevo3100 (Fujifilm VisualSonics, Canada). M-mode ultrasound and Doppler ultrasound modes were used to evaluate left ventricular systolic and diastolic function, including ejection fraction (LVEF), Early diastolic transmitral flow velocity/Atrial contraction-induced transmitral flow velocity (E/A) ratio, early mitral inflow velocity/Early diastolic mitral annular velocity (E/E′) ratio, and Isovolumic Relaxation Time (IVRT). All data were analyzed using Vevo Lab software (Fujifilm VisualSonics, Canada).

### Cardiac pressure-volume measurement

Mice were anesthetized with intraperitoneal injection of tribromoethyl aicolaol and then fixed on the operating table (AD Instruments,PL2604). The trachea was exposed and connected to a ventilator to maintain breathing. The left carotid artery was then isolated and a Millar catheter was inserted into the left ventricle. Different fluids were injected and the left ventricular pressure-volume curve was recorded. Finally, parameters such as left ventricular pressure and heart rate were analyzed to assess cardiac function.

### Sirius red staining

According to the standard protocol, the cardiac tissue paraffin sections were stained with the Sirius Red dye (Servicebio, G1018). Subsequently, the sections were observed using a confocal microscope (Nikon) to record the distribution and morphology of the collagen fibers.

### DHE staining

According to the standard protocol, the cardiac sections were stained with dihydroethidium (DHE, Biotime, S0063) and DAPI (1 μg/mL) and then observed and photographed using a confocal microscope.

### Oral glucose tolerance test (OGTT) and insulin tolerance test (ITT)

The experiment began after C57BL/6N mice were fasted for 12 h (water was not allowed). Blood was collected from the tail vein to measure basal blood glucose (0 min). Mice were then intraperitoneally injected with 20% glucose solution (2 g/kg). Blood was collected from the tail vein 15, 30, 60, 90, and 120 min after injection. Blood glucose levels were measured using blood glucose test strips (ACCU-CHEK, 06454011). The area under the curve (AUC) was calculated to assess glucose tolerance in the mice.

To maintain the consistency of the fasting condition in mice. C57BL/6N mice were fasted for 12 h (water was not allowed) and then given a small amount of chow. The experiment began after another 6 h of fasting. Blood was collected from the tail vein to measure basal blood glucose (0 min). Immediately afterward, 0.5 U/kg insulin solution (HTBT, G202505047) was intraperitoneally injected. Blood was collected from the tail vein 15, 30, 60, and 120 min after injection. The area under the curve (AUC) was calculated to assess the mice’s insulin sensitivity.

### Quantitative polymerase chain reaction (qPCR)

The qPCR was performed according to the standard protocol with CFX Connect™ Fluorescence quantitative PCR detection system (BIO-RAD). The primers used as follows: Mouse SOCS3-F: ATG​GTC​ACC​CAC​AGC​AAG​TTT; Mouse SOCS3-R: TCC​AGT​AGA​ATC​CGC​TCT​CCT; Mouse SOCS3-F: TGC​AGG​AAG​AAA​CCG​TTG​GAG; Mouse SOCS3-R: CTC​GTT​TTA​GGA​CTG​GAC​ACT​TG; Mouse IL-1β-F:TGCCACCTTTTGACAGTGATG; Mouse IL-1β-R:TGATGTGCTGCTGCGAGATT; Mouse IL-6-F:TAGTCCTTCCTACCCCAATTTCC; Mouse IL-6-R:TTGGTCCTTAGCCACTCCTTC; Mouse TNF-α-F:CCCTCACACTCAGATCAT CTTCT; Mouse TNF-α-R:GCTACGACGTGGGCTACAG; Mouse GAPDH-F:TGCGACTTCAACAGCAACTC; Mouse GAPDH-R:ATGTAGGCAATGAGGTCCAC. The results were exported in excel format. Then, “internal control correction” was performed on the target gene, followed by “control correction” for the experimental group. The difference between the two steps is ^△△^CT. Finally, the CT difference was linearly transformed to the expression fold by 2^(^-△△^CT).

### Western blotting

Western blotting was used to assess cardiac insulin resistance in mice using standard protocols. Antibodies against IRS1-Ser307 (Proteintech, 85238-1-RR, dilution 1:5000), P-AKT1-T308+AKT2-T309+AKT3-T305 (ABclonal, AP1266, dilution 1:800), SOCS3 (Proteintech, 66797-1-Ig, dilution 1:20,000), and GAPDH (Servicebio, GB15002-100, dilution 1:8000) were used.

### GLUT4 staining

After dewaxing and rehydration with gradient ethanol of the mouse heart paraffin sections (5 µM), they were treated with 0.1% Triton X-100 for 10 min and 1% BSA for 30 min for blocking. The sections were incubated with GLUT4 antibody (Proteintech, 66846-1-Ig) at 4 °C overnight; washed with PBS 3 × 5 min, incubated at room temperature in the dark with fluorescent secondary antibody for 1 h. Washed with PBS 3 × 5 min again, then added fluorescent WGA (Thermo W11261) in the dark for 30 min, sealed with DAPI and photographed under a fluorescence microscope.

### mRNA sequencing

The bulk RNA-seq of heart tissue was performed in accordance with established protocols (Shi et al., 2022). In brief, heart tissues were collected after perfusion with old PBS. Atrial and connective tissue were promptly excised, and the remaining ventricular tissues were rapidly frozen in liquid nitrogen and stored at −80 °C until further processing. The samples were then sent to OE Biotech Co., Ltd. (Shanghai, China) for paired-end sequencing (PE150) on an Illumian Novaseq™ 6000. After sequencing and quality control, the clean reads were aligned to the mm10 genome using HISAT2 (https://daehwankimlab.github.io/hisat2/), and quantification was performed with HTSeq-count (Anders et al., 2015). Differential gene expression analysis was executed using DEseq2, and matrix normalized expression matrix was generated using the variance stabilizing transformation (VST) function of DEseq2 (Love et al., 2014).

### Mfuzz cluster analysis

The Mfuzz package (version 2.64.0), which employs fuzzy c-means clustering, was utilized to perform cluster analysis on the matrix of normalized valuse derived from bulk RNA-seq data (Gao et al., 2017). This analysis aimed to categorize genes into distinct clusters. Post-cluster analysis, clusters that did not demonstrate a significant opposing trend between HF_vs._NC and HFi_vs._HF were manually excluded. The genes within the retained clusters underwent further analysis using the Mfuzz package. Ultimately, after three iterations of Mfuzz analysis, the results were obtained.

### Statistical analysis

Data were analysed by SPSS (Version 26.0, IBM SPSS Statistics). Normality was tested using the Shapiro-Wilk method. For differences between two independent groups, parametric tests (independent t-test) and nonparametric tests (Mann-Whitney U test) were used based on the normal distribution of the data. When statistical analysis involved three or more groups, one-way analysis of variance (ANOVA) Tukey’s *post hoc* test was used for normally distributed data, and the Kruskal–Wallis test was used for non-normally distributed data. All statistical analyses were performed using SPSS. Data are showed as mean ± standard deviation, and P < 0.05 was considered statistically significant. Statistical analysis and drawing were performed using the GraphPad Prism 8 (GraphPad, San Diego, California, United States). Analysis of correlation by simple linear regression model. Significant among groups was analyzed by One-way ANOVA. Unpaired t-test was used for comparing depending on whether the samples are paired. A two-sided P value <0.05 was considered statistically significant.

## Results

### Synthesis and characterization of Mn–ZIF nanozymes

**FIGURE 1:**
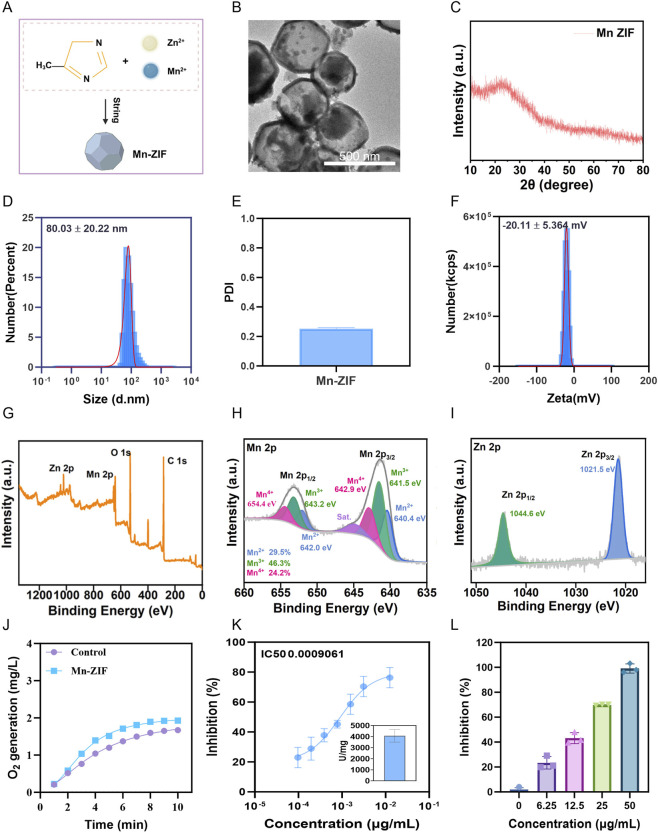
Structural design and physicochemical characterization of Mn-ZIF nanozyme. **(A)** Schematic illustration of Mn-ZIF nanozyme synthesis. **(B)** TEM images of Mn-ZIF nanozyme. Scale bar = 500 μm. **(C)** X-ray diffraction (XRD) pattern of the Mn-ZIF nanozyme. **(D)** Hydrodynamic diameters of Mn-ZIF. **(E)** The polydispersity index of Mn-ZIF nanozyme (n = 3). **(F)** Zeta potentials of Mn-ZIF. **(G)** Elemental composition of Mn-ZIF nanozymes determined by XPS. **(H)** High-resolution XPS spectra of Mn 2p for Mn-ZIF nanozyme. **(I)** High-resolution XPS spectra of Zn 2p for Mn-ZIF nanozyme. **(J)** Dissolved oxygen generation of Probiotics and BPEP in 200 mM H_2_O_2_ solution (n = 3). **(K)** SOD-like activity evaluation of Mn-ZIF nanozyme (n = 3). **(L)** Concentration-dependent ABTS radical scavenging efficiency of Mn-ZIF nanozyme (n = 3).

Mn–ZIF nanozymes were synthesized through a coordination–self-assembly–in situ metal doping strategy (Figure 1A). Briefly, dopamine, Zn(NO_3_)_2_·6H_2_O, and Mn (NO_3_)_2_·6H_2_O were dissolved in deionized water to form solution A, while 2-methylimidazole was dissolved in 9 mL of deionized water to form solution B. Subsequently, solution A was rapidly poured into solution B under magnetic stirring at room temperature and stirred for 12 h to complete the coordination and self-assembly process. The resulting suspension was centrifuged and washed three times with deionized water, followed by freeze-drying to obtain brown Mn–ZIF powders.

Transmission electron microscopy (TEM) revealed that the obtained Mn–ZIF particles exhibited a uniform polygonal morphology with good monodispersity (Figure 1B). X-ray diffraction (XRD) showed characteristic peaks identical to those of ZIF-8, with no additional diffraction peaks, confirming that the crystalline topology of ZIF-8 was preserved after Mn doping (Figure 1C). Dynamic light scattering (DLS, Figures 1D,E) analysis indicated a hydrodynamic diameter of 80 ± 20 nm with a low polydispersity index (PDI <0.30) (Figures 1D,E). The zeta potential was measured as −20.11 ± 5.36 mV, suggesting excellent colloidal stability (Figure 1F). X-ray photoelectron spectroscopy (XPS) revealed the coexistence of Mn 2p3/2 (641.5 eV) and Mn 2p1/2 (643.2 eV) peaks, as well as Zn 2p3/2 (1021.5 eV) and Zn 2p1/2 (1044.6 eV) peaks, verifying the successful coordination of Mn^2+^ and Zn^2+^ ions within the ZIF framework without structural disruption (Figures 1G–I). Catalytic activity evaluation demonstrated that Mn–ZIF generated 9 mg/L dissolved oxygen within 10 min in 200 mM H_2_O_2_ (Figure 1J), inhibited 50% of superoxide anion radicals at a concentration of 0.0009601 μg/mL (Figure 1K), and exhibited strong ABTS radical scavenging activity across various concentrations (6.25, 12.5, 25, and 50 μg/mL) (Figure 1L). These results indicate that Mn–ZIF possesses integrated superoxide dismutase (SOD)-, catalase (CAT)-, and radical scavenging–like activities, providing a robust structural and functional basis for subsequent *in vivo* antioxidant applications.

To achieve the cardiac targeting ability of Mn–ZIF nanozyme, atrial natriuretic peptide (ANP) was modified on the surface of Mn–ZIF to enhance its cardiac targeting property. As shown in Supplementary Figure S1, the bare Mn-ZIF nanozyme exhibited a zeta potential of approximately −23 mV, consistent with its inherent surface properties. Upon coating with a neutral lipid layer (without ANP) to form NanoM, the surface potential shifted to about −8 mV. This significant change confirms the successful formation of a lipid shell, which effectively masks the original surface charge of the core. Following the subsequent incorporation of ANP to yield the final NanoAM (Mn–ZIF nanozyme is modified by ANP), the zeta potential showed a further, distinct shift to approximately −10 mV. The sequential and consistent changes in surface potential strongly support the successful integration of ANP into the nanoparticle structure.

### ROS scavenging by NanoAM

**FIGURE 2:**
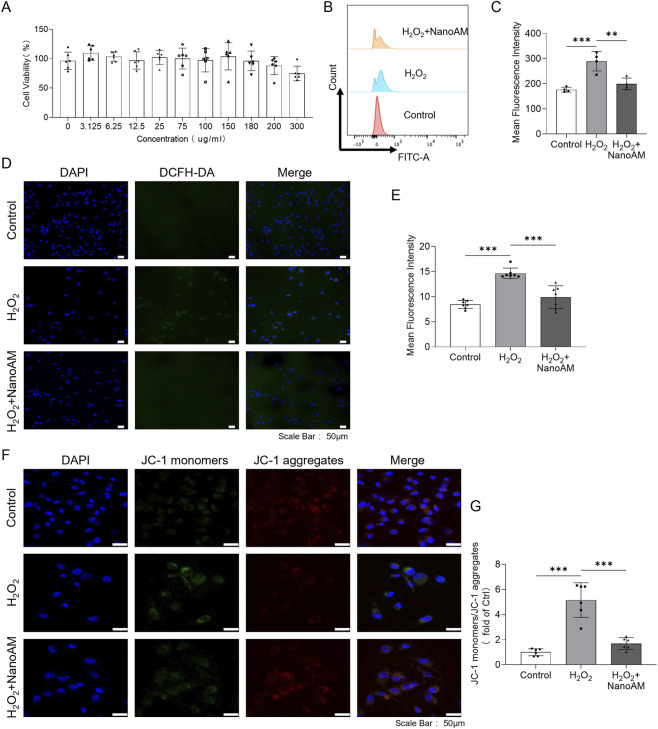
ROS scavenging by NanoAM. **(A)** CCK8 assays were used to detect the cytotoxicity of NanoAM in different concentrations. **(B–G)** AC16 cells were pretreated with 15 mg/mL NanoAM for 6 h and then induced by H_2_O_2_ for 24 h, the ROS level was evaluated by flow cytometry analysis **(B,C)** and. DCFH-DA probe **(D,E)**, Mitochondrial membrane potential was assessed using JC-1 staining **(F,G)**. Scale bar, 50 μm. All experiments were performed with at least three biological replicates. Data are presented as mean ± SD and analyzed using one-way ANOVA with Tukey’s *post hoc* test; **p < 0.01, ***p < 0.001.

To evaluate the biocompatibility of the material, CCK8 assay was conducted and the results showed that cell viability remained above 70% across all NanoAM -treated groups (0–300 μg/mL), indicating that the material has ideal safety (Figure 2A). Flow cytometry analysis revealed a rightward shift in the fluorescence peak in the H_2_O_2_-treated group compared to the Control, whereas the peak in the H_2_O_2_ + NanoAM group closely resembled that of the Control (Figures 2B,C). The DCFH-DA assay further supported these findings, with elevated fluorescence intensity in the H_2_O_2_ group (∼1.75 fold) that was markedly attenuated by NanoAM treatment (Figures 2D,E). The JC-1 assay showed a rise in JC-1 monomer fluorescence and a reduction in JC-1 aggregates upon H_2_O_2_ exposure, both of which were mitigated by NanoAM (Figures 2F,G). Collectively, these results demonstrate the potent ROS scavenging capacity of NanoAM.

### NanoAM exhibits ideal cardiac targeting and biocompatibility

**FIGURE 3:**
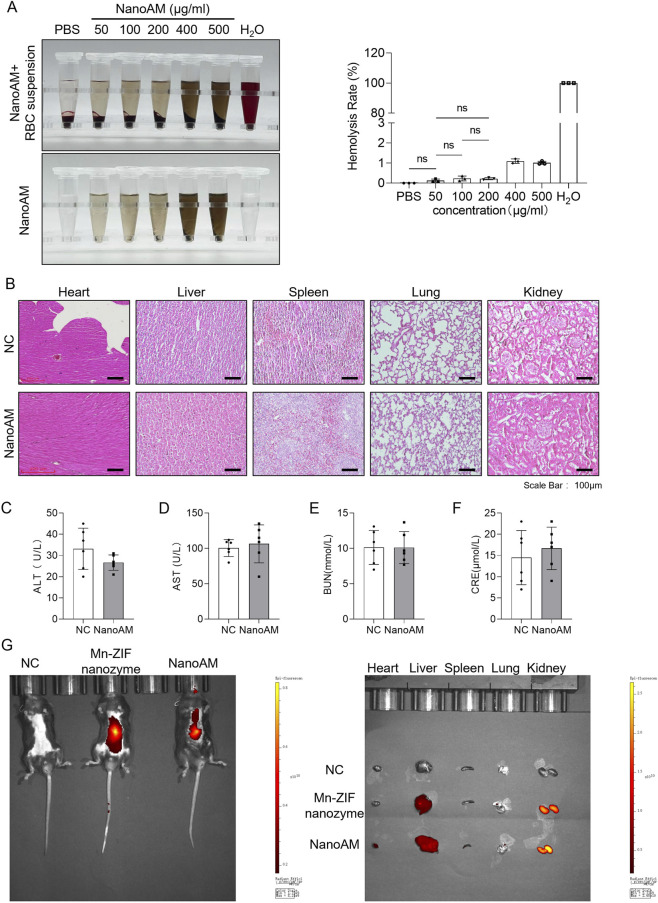
NanoAM exhibits ideal cardiac targeting and biocompatibility. **(A)** Hemolysis assay of NanoAM at various concentrations., PBS served as a negative control, and distilled water served as a positive control; **(B–F)** C57BL/6N mice were injected with 15 mg/kg NanoAM (twice weekly) for 12 weeks. H&E staining was used to observe the effects of NanoAM on the heart, liver, spleen, lung, and kidney of mice. Scale bar: 100 μm **(B)**. ALT, AST, BUN and CRE were measured to evaluate the effect of NanoAM on the liver and kidney function **(C–F)**; **(G)** *In vivo* distribution of CY5.5-labeled NanoAM (15 mg/kg)after 6 h injection, as detected by *in vivo* imaging. (Data are showed as mean ± SD and analyzed using one-way ANOVA with Tukey’s *post hoc* test; ns, no statistically significant difference).

To confirm that NanoAM could accumulate effectively in the heart without inducing systemic toxicity, we first assessed its hemocompatibility. Hemolysis assays revealed that the hemolysis rate remained within a safe range (<5%) at concentrations up to 200 μg/mL (Figure 3A). Subsequently, mice were administered 15 mg/kg NanoAM via tail vein injection twice a week for 12 weeks. Histological examination by H&E staining showed no obvious pathological changes in the heart, liver, spleen, lung, or kidney (Figure 3B). Furthermore, no significant differences were observed in key biochemical markers of liver and kidney function between NanoAM-treated and untreated mice (Figures 3C–F), indicating minimal adverse effects on organ function. These results demonstrate the good biocompatibility of NanoAM.

We next investigated the cardiac targeting capability of NanoAM using a small-animal *in vivo* imaging system. 15 mg/kg NanoAM was injected into the tail vein twice a week for 2 weeks. NanoAM showed significant accumulation in the heart, whereas control particles without NanoAM did not exhibit cardiac targeting (Figure 3G). This confirms that NanoAM possesses specific affinity for cardiac tissue.

### NanoAM alleviates heart dysfunction and hypertension in HFpEF mice

**FIGURE 4:**
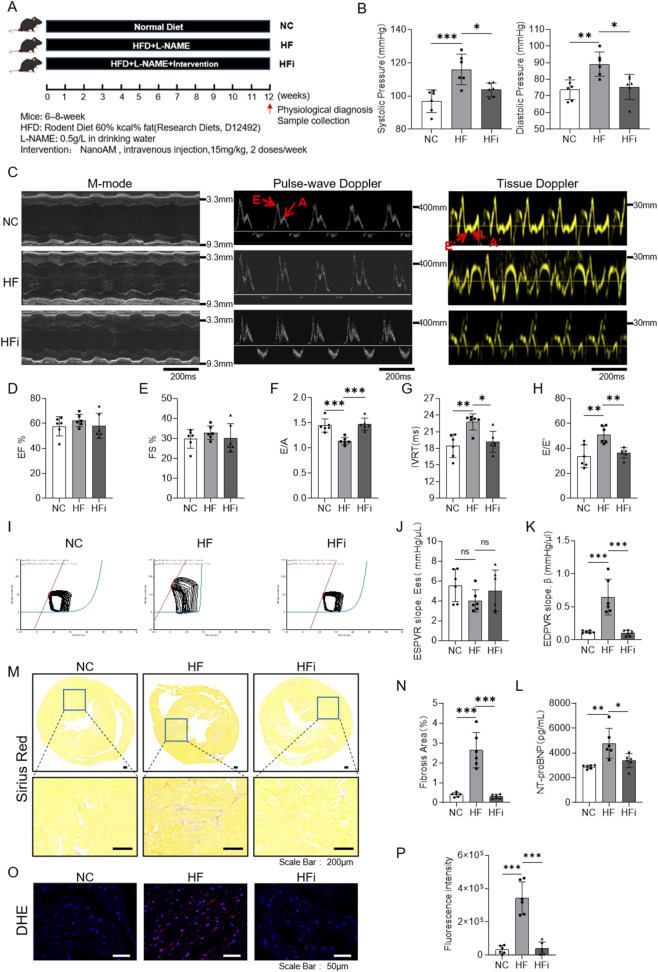
NanoAM alleviates heart dysfunction and hypertension in HFpEF mice. **(A)** Schematic diagram of the animal experiment. A total of 18 mice were randomly divided into three groups (n = 6). NC (standard diet), HFpEF (high-fat diet + L-NAME), and HFi (HFpEF diet +15 mg/kg NanoAM via tail vein, twice weekly) **(B)** Systolic and diastolic blood pressure were measured by non-invasive rat tail blood pressure measurement system. **(C–H)** Echocardiographic parameters: EF, FS, E/A, IVRT, and E/E′ (n = 6 per group). **(I-K)** Cardiac pressure-volume loops were analyzed, and the results for ESPVR and EDPVR are presented in figure J and K (n = 6). **(L)** Serum NT-proBNP level were measured by ELISA (n = 6). **(M,N)** Heart Sirius Red staining and analysis were shown. Scale bar: 200 μm. **(O,P)** Cardiac ROS staining and fluorescence intensity quantification (n = 6). Data are presented as mean ± SD and were analyzed using one-way ANOVA followed by Tukey’s *post hoc* test; *p < 0.05, **p < 0.01, ***p < 0.001, ns: not significant.

To evaluate the therapeutic potential of NanoAM in HFpEF, 18 mice were randomly divided into three groups: normal control (NC, normal chow and water), heart failure (HF, high-fat diet [HFD; 60% kcal fat, D12492] and 0.5 g/L L-NAME in drinking water for 12 weeks), and HF with NanoAM intervention (HFi, HFD + L-NAME + intravenous NanoAM at 15 mg/kg, twice weekly) (Figure 4A). Non-invasive tail blood pressure measurement system showed that systolic blood pressure increased from 95.2 mmHg (NC group) to 115.2 mmHg in HF group (p < 0.001), and decreased to 104.6 mmHg after NanoAM treatment (p < 0.05). Diastolic blood pressure increased from 69.7 mmHg (NC group) to 88 mmHg in HF group (p < 0.01), and was reduced to 77 mmHg following NanoAM administration (p < 0.05) (Figure 4B). After 12 weeks of HFD + L-NAME feeding, mice developed a clinical HFpEF-like phenotype: echocardiography revealed no significant differences in left ventricular ejection fraction (EF) and fractional shortening (FS) among the NC, HFpEF, and HFi groups (Figures 4C–E). And the E/A ratio decreased from 1.4 (NC) to 1.15 in HF mice (p < 0.001), and recovered to 1.5 with NanoAM treatment (Figure 4F). The IVRT increased from 20 ms (NC) to 25.2 ms in the HF group (p < 0.01), and decreased to 18.7 ms after intervention (p < 0.05) (Figure 4G). The E/E′ ratio also increased from 33 (NC) to 47.2 in the HF group, and declined to 36.7 post-treatment (p < 0.01) (Figure 4H). Pressure-volume (PV) loops analysis indicated preserved systolic function but impaired diastolic function in the HF group, evident as an upward shift of the PV loop (Figure 4I). While end-systolic PV relationship (ESPVR) slope Ees did not differ among groups (Figure 4J), the end-diastolic PV relationship (EDPVR) slope β increased from 0.1 mmHg/μL (NC) to 0.7 mmHg/μL, and returned to 0.11 mmHg/μL after NanoAM administration (p < 0.001) (Figure 4K). The serum NT-proBNP level in the NanoAM group was significantly lower than that in the HF group (3383.7 pg/ml vs 4777.3 pg/ml) (Figure 4L). Sirius red staining revealed increased collagen deposition (red) in HF mice (2.6%) compared to NC (0.4%), which was ameliorated by NanoAM (0.3%) (p < 0.001) (Figures 4M,N). DHE staining showed elevated ROS level in HF heart (∼5.5 fold) compared to NC (p < 0.001), which was mitigated by NanoAM (∼1.12 fold) (Figures 4O,P). In addition, although there was no significant difference in body weight between the HFi group and the HF group, the heart weight/tibia length ratio (HW/TL) was statistically significantly decreased in the HFi group (Supplementary Figures S2A, B). In sum, NanoAM alleviates heart dysfunction and hypertension in HFpEF mice.

### NanoAM ameliorates HFpEF by modulating the insulin resistance pathway via SOCS3

**FIGURE 5:**
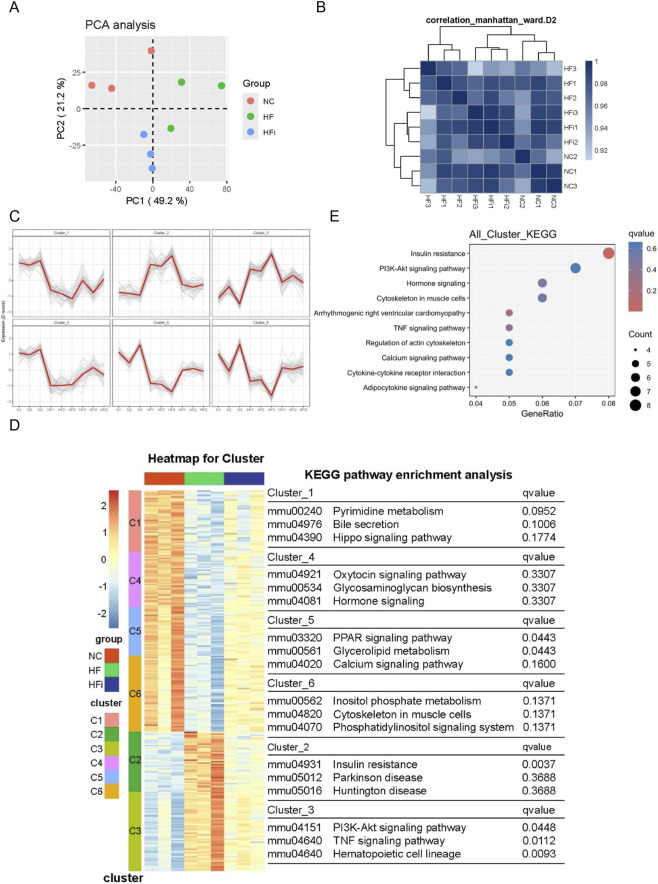
NanoAM attenuates insulin resistance pathway activation in HFpEF hearts. **(A)** Principal component analysis and **(B)** correlation heatmap illustrate the differences among samples from different groups. **(C)** Six gene clusters with similar expression patterns were clustered using the Mfuzz R package. **(D)** The gene expression heatmap and KEGG function annotation for the six clusters were illustrated. **(E)** KEGG analysis of all clusters was demonstrated.

To further investigate the molecular mechanisms underlying the effect of NanoAM on HFpEF, we performed bulk RNA-seq on cardiac tissues from normal control (NC), HFpEF (HF), and NanoAM-treated HFpEF (HFi) groups. Principal component analysis (PCA) (Figure 5A) and sample correlation analysis (Figure 5B) demonstrated good intra-group consistency, reflecting high data quality. To identify genes potentially reversed by NanoAM treatment, we focused on genes that were differentially expressed in HF vs. NC and showed opposite expression trends in HFi vs. HF. A total of 1165 differentially expressed genes (DEGs) were identified between HF and NC, including 663 upregulated and 502 downregulated genes (Supplementary Table S1). Gene co-expression analysis was performed using the Mfuzz packages in R studio. After multiple rounds of filtering, six gene clusters with coherent expression patterns were identified (Figure 5C; Supplementary Figure S3) (Supplementary Table S2). Cluster 1, 4, 5, and 6 comprised genes upregulated in HF and downregulated in HFi, while cluster 2 and 3 contained genes downregulated in HF and upregulated in HFi. KEGG pathway analysis for each cluster revealed significant enrichment of metabolism-related pathways, such as PPAR signaling, glycerolipid metabolism, and insulin resistance in cluster 2 and 5. The PI3K-Akt signaling pathway and the inflammation-related TNF signaling pathway were markedly enriched in cluster 3 (Figure 5D) which are known to be involved in cardiac dysfunction. Integrated KEGG analysis of all co-expressed genes further confirmed significant enrichment of the insulin resistance, PI3K-Akt signaling, and TNF signaling pathways, with insulin resistance being the most prominently enriched (Figure 5E).

**FIGURE 6:**
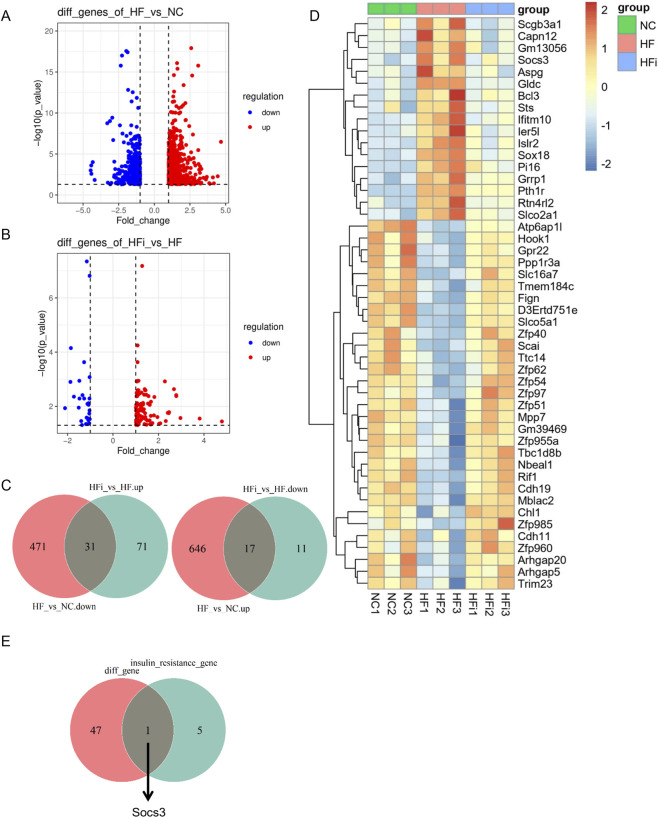
*Socs3* expression is elevated in HFpEF hearts and reduced after NanoAM treatment. **(A,B)** Volcano plots illustrate differential gene expression between groups: HF versus NC **(A)**, and HFi versus HF **(B,C)** Venn diagrams display the number of genes with opposite expression patterns in the HFi versus HF comparison relative to the HF versus NC comparison. **(D)** A Heatmap presents the relative expression levels of the 48 genes across three groups. **(E)** Interaction network between the 48 genes and insulin resistance (IR)-related genes enriched in Figure 5D, identifying *Socs3* as the key overlapping gene.

We further investigated which specific genes were reversed by NanoAM. A total of 130 DEGs were identified between HFi and HF, including 102 upregulated and 28 downregulated genes (Supplementary Table S3). By integrating these with the DEGs from the HF vs. NC comparison (Figures 6A,B), we identified 48 genes, of which 31 genes that were downregulated in HF vs. NC and upregulated in HFi vs. HF; and 17 genes that were upregulated in HF vs. NC and downregulated in HFi vs. HF (Figures 6C,D). Among these, only *Socs3* overlapped with the insulin resistance pathway genes identified in Figure 5E (*Gfpt2*, *Slc27a3*, *Socs3*, *Trib3*, *Rps6kal*, *Tnfrsfla*) (Figure 6E). *Socs3* was upregulated in HF compared to NC and downregulated after NanoAM treatment.

### NanoAM ameliorates insulin resistance via regulating the SOCS3-IRS1-AKT2 axis

**FIGURE 7:**
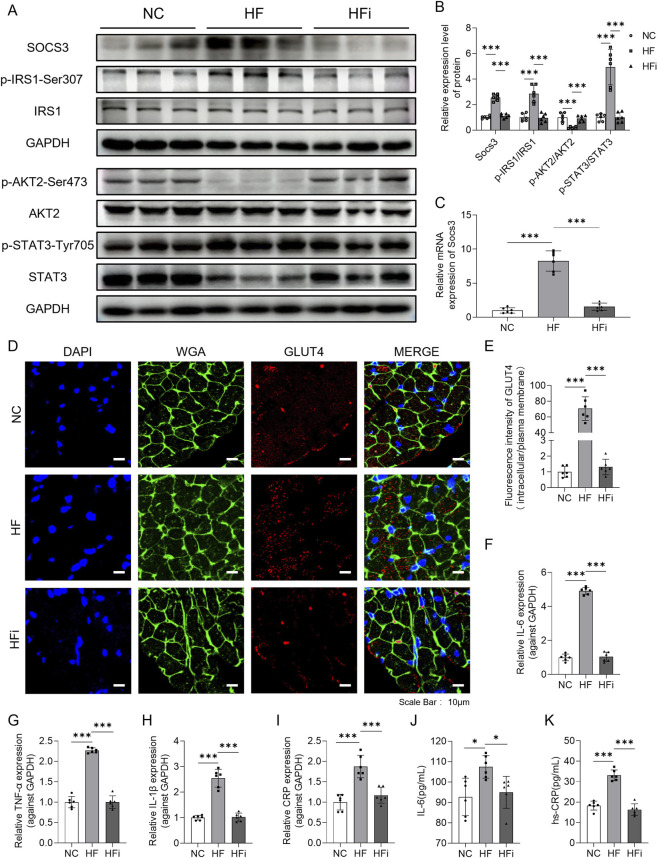
NanoAM regulates the SOCS3-IRS1-AKT2 axis to improve insulin resistance. **(A,B)** WB analysis of insulin resistance-related proteins in heart tissue (n = 6). **(C)** The *Socs3* mRNA expression levels in heart tissues were determined by qPCR. **(D)** Immunofluorescence staining of GLUT4 in cardiac tissues. Scale bar, 10 µm. **(E)** Fluorescence intensity quantification of GLUT4 was shown. **(F–I)** The Inflammatory factors (IL-6,TNF-α,IL-1β,CRP) mRNA expression levels in heart tissues were determined by qPCR. **(J,K)** Serum levels of IL-6 and hs-CRP measured by ELISA. Data are showed as mean ± SD and analyzed using one-way ANOVA followed by Tukey’s *post hoc* test; *p < 0.05, ***p < 0.001.

*Socs3*, a key suppressor of cytokine signaling, is induced by inflammatory cytokines such as IL-6 and IL-10 (Gao et al., 2018) and inhibits insulin signaling by targeting IRS1 (Rui et al., 2002). Western blot analysis revealed that NanoAM downregulated SOCS3 protein expression, reduced phosphorylation of IRS1 at Ser307, enhanced AKT2 phosphorylation, and promoted downstream insulin signaling (Figures 7A,B). We further evaluated *Socs3* expressions among NC, HF and HFi groups using qPCR. The qPCR results further showed that *Socs3* mRNA levels were significantly increased in HFpEF hearts (10.7-fold vs. NC, p < 0.001) and decreased after NanoAM treatment (2.3-fold) (Figure 7C). Immunofluorescence staining of heart sections showed that NanoAM promoted GLUT4 translocation from the cytoplasm to the membrane, indicating improved glucose uptake (Figures 7D,E). Then we evaluated inflammatory factors (IL-6, TNF-α, IL-1β, CRP) expression among NC, HF and HFi groups using qPCR. Results showed that these target genes’ mRNA levels were significantly increased in HFpEF hearts and decreased after NanoAM treatment (Figures 7F–I). Meanwhile, serum IL-6 and high-sensitivity C-reactive protein (hs-CRP) levels were significantly elevated in HFpEF mice and effectively alleviated after NanoAM treatment (Figures 7J,K).

## Discussion

Heart failure with preserved ejection fraction (HFpEF) has become a major challenge in cardiovascular disease, characterized by high heterogeneity, complex pathological mechanisms, and a lack of effective therapeutic strategies (Li et al., 2025). Increasing evidence has suggested that excessive reactive oxygen species (ROS) generation and oxidative stress seem to play a critical role in the pathogenesis of HFpEF (Mishra and Kass, 2021; Schiattarella et al., 2019). Small amounts of ROS are essential for maintaining mitochondrial homeostasis and cardiac function, while excessive ROS production is often associated with detrimental cardiac remodeling (Mongirdienė et al., 2022). Furthermore, it has been found that oxidative stress is elevated in both HFrEF and HFpEF and is associated with the pathogenesis of myocardial remodeling; however, the related downstream pathways differ between them (Mongirdienė et al., 2022). Therefore, drug-targeted specific ROS and the pathways they induce may be beneficial for heart failure patients, including HFpEF. In this study, we developed a cardiac-targeting ROS-scavenging nanozyme, which can effectively accumulate in cardiac tissue to selectively eliminate ROS, mitigate oxidative stress and inflammation, and improve diastolic dysfunction along with related pathological phenotypes. Furthermore, we discovered that its therapeutic efficacy against HFpEF may be achieved through a novel ROS-inflammation-insulin resistance axis, thereby demonstrating dual therapeutic effects.

Inspired by the catalytic mechanism of natural manganese superoxide dismutase (Mn-SOD), we rationally designed a Mn–ZIF nanozyme that structurally mimics the Mn-SOD active center by coordinating Mn^2+^ with nitrogen-rich imidazole ligands within a confined porous framework. This biomimetic configuration provides a microenvironment analogous to the enzymatic pocket of native Mn-SOD, facilitating efficient electron transfer and redox cycling between Mn^2+^/Mn^3+^ during catalytic reactions. As a result, the Mn–ZIF nanozyme exhibited potent superoxide dismutase–like activity of approximately 4000 U/mg, effectively catalyzing the dismutation of superoxide anions into molecular oxygen and hydrogen peroxide. Such high catalytic efficiency not only confirms the successful structural mimicry of the Mn-SOD active site but also underscores the potential of Mn–ZIF as a robust antioxidant nanozyme for combating excessive ROS in pathological cardiac remodeling.

Pharmacological intervention for heart disease is often limited by the high blood flow velocity in the cardiac region, which may reduce drug-tissue interactions, thereby diminishing therapeutic efficacy (Genç et al., 2022). Developing nanozyme-based drugs with cardiac targeting capability is an important direction. Current strategies focus on modifying nanozymes with tannic acid to achieve cardiac-targeted accumulation (Gu et al., 2024; Xing et al., 2025). In this study, based on our previous work (Shin et al., 2018), we have achieved excellent cardiac-targeting efficacy by modifying nanozymes with atrial natriuretic peptide (ANP) antibodies. Compared to tannic acid, which relies on hydrogen bonding or coordination interactions between its polyphenol groups and cardiac tissue components such as elastin and collagen (Haass et al., 2011), ANP antibody modification enables direct anchoring of the nano-drug to cardiomyocytes via antigen-antibody interactions with ANP, which is highly expressed under pathological conditions. This approach may offer advantages in terms of tissue specificity and retention. Through *in vitro* CCK-8 assays, hemolysis tests, and long-term detection of histopathological and biochemical indices in heart failure with preserved ejection fraction (HFpEF) mice, it was confirmed that NanoAM can achieve specific accumulation in the heart, thereby minimizing off-target effects and exhibiting good biocompatibility and safety. Furthermore, NanoAM can effectively improve cardiac diastolic dysfunction in HFpEF mice, as evidenced by improvements in cardiac ultrasound and pressure-volume indices, reduced blood pressure, alleviated myocardial hypertrophy, and attenuated myocardial fibrosis.

To further clarify the mechanism by which NanoAM improves cardiac diastolic function in HFpEF mice, we used bulk RNA sequencing technology to analyze the gene expression profiles of the cardiac tissues of mice. We observed significant enrichment and upregulation of signaling pathways related to insulin resistance, PI3K/AKT, and inflammation (specifically TNF) in the cardiac tissues of HFpEF mice. Among these, the insulin resistance pathway showed the most prominent enrichment. However, treatment with NanoAM significantly reduced the expression of genes associated with these pathways. HFpEF is associated with a variety of metabolic comorbidities, such as obesity, diabetes, and hypertension (Li et al., 2025; Paneni et al., 2013). Among these comorbidities, insulin resistance plays a key role in the interaction between metabolic disorders and HFpEF (Paneni et al., 2013; Capone et al., 2022), and significantly impairs cardiomyocyte function (Capone et al., 2023; Hahn et al., 2023). These comorbidities can promote a systemic pro-inflammatory state through various mechanisms (Paneni et al., 2013). Existing studies have shown that inflammation and oxidative stress usually occur in an interdependent manner (Mittal et al., 2014). Clinically, elevated levels of IL-6, TNF-α, and ROS are frequently observed in HFpEF patient (Bode et al., 2020). Through serological and tissue-level detection, we found that the IL-6 level was significantly increased in HFpEF mice, while NanoAM treatment significantly reduced the IL-6. Meanwhile, the results of immunofluorescence, GTT, and ITT further confirmed that NanoAM treatment significantly improved insulin resistance and glucose metabolism in HFpEF mice. These findings suggest that beyond its inherent ROS-scavenging ability, NanoAM may exert synergistic therapeutic effects on cardiac function in HFpEF mice by ameliorating systemic inflammation and modulating the insulin resistance signaling pathway.

Furthermore, to explore the potential mechanism by which NanoAM improves insulin resistance, we performed an integrated analysis of RNA sequencing data. The results revealed that SOCS3, a key suppressor of cytokine signaling, participates to the insulin resistance pathway and was significantly upregulated in HFpEF mice, while its expression was downregulated in both normal and NanoAM-treated mice. Therefore,we hypothesize that SOCS3 may be a critical gene in this regulatory process. SOCS3 is known to be induced by IL-6 via STAT3 activation (Kim et al., 2008). Under physiological conditions, SOCS3 provides negative feedback to prevent excessive IL-6 signaling, thereby maintaining cellular signal homeostasis (Croker et al., 2003). However, SOCS3 overexpression can bind to IRS1 and promote its ubiquitination and degradation, leading to reduced insulin sensitivity and exacerbation of insulin resistance-related pathologies (Rui et al., 2002; Xu et al., 2020). As a limitation of this study, we will also continue to investigate the interaction between SOCS3 and IRS1, as well as the potential mechanism by which this interaction leads to ubiquitination and degradation of IRS1, ultimately contributing to the development of HFpEF.

The PI3K/Akt (phosphatidylinositol 3-kinase/protein kinase B) pathway is one of the critical pathways in insulin signaling (Medina-Vera et al., 2021). Upon activation, IRS1 undergoes tyrosine phosphorylation, which further activates the PI3K/AKT signaling pathway. Activated AKT then phosphorylates its substrate AS160 (Akt substrate 160), thereby relieving the inhibition on glucose transporter 4 (GLUT4) (Sharma and Dey, 2021). This promotes the translocation of GLUT4 from the cytoplasm to the cell membrane, enabling it to exert its glucose uptake function (Ahmad, 2025). Western blot and immunofluorescence result further confirmed the activation of this signaling pathway. Therefore, we propose that in addition to its intrinsic ROS-scavenging capacity, NanoAM can also enhance cellular glucose uptake capacity by activating the SOCS3-IRS1-AKT2 signaling axis. This process improves insulin sensitivity and ameliorates the HFpEF phenotype.

## Conclusion

In this study, we developed a nanozyme NanoAM, which is modified by ANP and possesses significant cardiac targeting capability. Validated by both *in vitro* and *in vivo* experiments, this nanozyme exhibits excellent cardiac targeting, biocompatibility, and safety. Moreover, it showed remarkable efficacy in improving cardiac diastolic dysfunction, lowering blood pressure, reducing cardiac fibrosis, and mitigating myocardial hypertrophy in HFpEF mice induced by a high-fat diet. However, this study also has certain limitations. When evaluating the role of NanoAM in regulating insulin resistance, we only assessed the systemic insulin resistance and glucose tolerance levels. A more rigorous approach would involve using the hyperinsulinemic-euglycemic clamp technique to detect localized cardiac insulin resistance. This method would more accurately reflect the nanozyme’s role in improving cardiac diastolic function by modulating insulin resistance-related pathways. This will also be an important direction for our future efforts to enhance the bioactivity of this nanozyme.

In summary, this novel nanozyme not only demonstrates its capacity to scavenge ROS but also suggests that it may exert dual therapeutic effects in the treatment of HFpEF by modulating insulin resistance signaling pathways. These findings provide new insights and potential intervention targets for the future clinical treatment of HFpEF.

## Data Availability

The datasets presented in this study can be found in online repositories. The names of the repository/repositories and accession number(s) can be found below: https://www.ncbi.nlm.nih.gov/, PRJNA1333645.
