# Supplementary material for: Bioinspired cardiac-targeted metal-organic framework nanozyme for modulating inflammatory responses in heart failure with preserved ejection fraction
Source: Front Bioeng Biotechnol. 2026 Feb 12;14:1744643. doi: 10.3389/fbioe.2026.1744643 (PMC12935870; doi:10.3389/fbioe.2026.1744643)
Supplement: Supplementary file 1 [file Supplementaryfile1.docx]

**Bioinspired** **Cardiac-Targeted Metal-Organic Framework** **Nanozyme for Modulating Inflammatory Responses in Heart Failure with Preserved Ejection Fraction**

Yuesheng Gui^1,^^2^, Xiaowan Fan^3^, Kairui Xiao^2^, Junyue Xing^2^, Zongfeng Niu^2^, Yingying Wang^2^, Weining Yuan^2^, Jia Shen^1,2^, Yingchao Shi^2^, Xiaolei Cheng^2^, Yu Han^2,*^, Zhen Li^2,4,*^, Hao Tang^1,2,4,*^.

1. Zhengzhou University People’s Hospital, Henan Provincial People’s Hospital, Zhengzhou, Henan, 450003, China

2. Zhengzhou Key Laboratory of Cardiovascular Aging, Henan Key Laboratory of Chronic Disease Management, Henan Province Key Laboratory for Prevention and Treatment of Coronary Heart Disease, National Health Commission Key Laboratory of Cardiovascular Regenerative Medicine, Central China Subcenter of National Center for Cardiovascular Hospital & Fuwai Central China Cardiovascular Hospital, Zhengzhou, Henan, 451464, China

3. School of Medicine of Henan University, Zhengzhou, 450046, Henan, China

4. Institute of Cardiovascular Disease, Henan Academy of Innovations in Medical Science, Zhengzhou, Henan 451162, China

**^*^**Correspondence: tangpku_zzuhao@zzu.edu.cn (H.T.), lizhen630@zzu.edu.cn (Z.L.), structure_han@zzu.edu.cn (Y.H.)


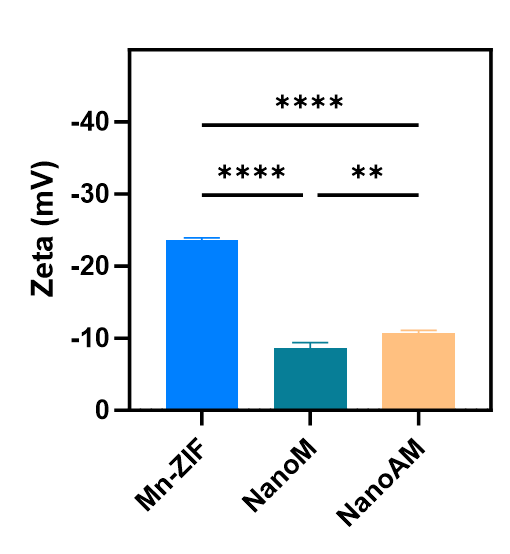


**Figure S1**. The Zeta of Mn-ZIF, NanoM, and NanoAM. Data are presented as mean±SD and were analyzed using one-way ANOVA followed by Tukey’s post hoc test; **p < 0.01, ****p < 0.0001.


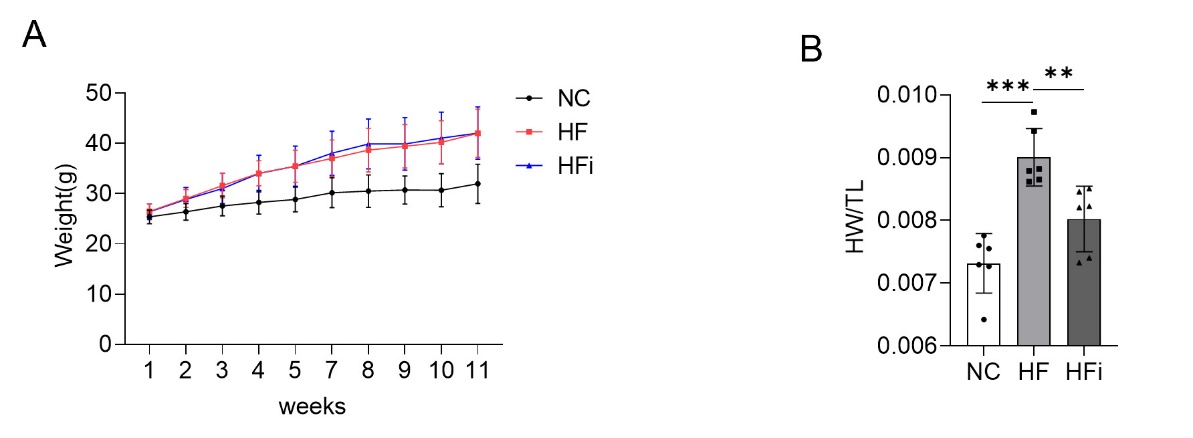


**Figure S2.**  (A) Body weight of mice in each group recorded every week (n=6). (B) Statistical results of the HW/TL ratios (n=6). Data are presented as mean±SD and were analyzed using one-way ANOVA followed by Tukey’s post hoc test; **p < 0.01, ***p < 0.001.

#


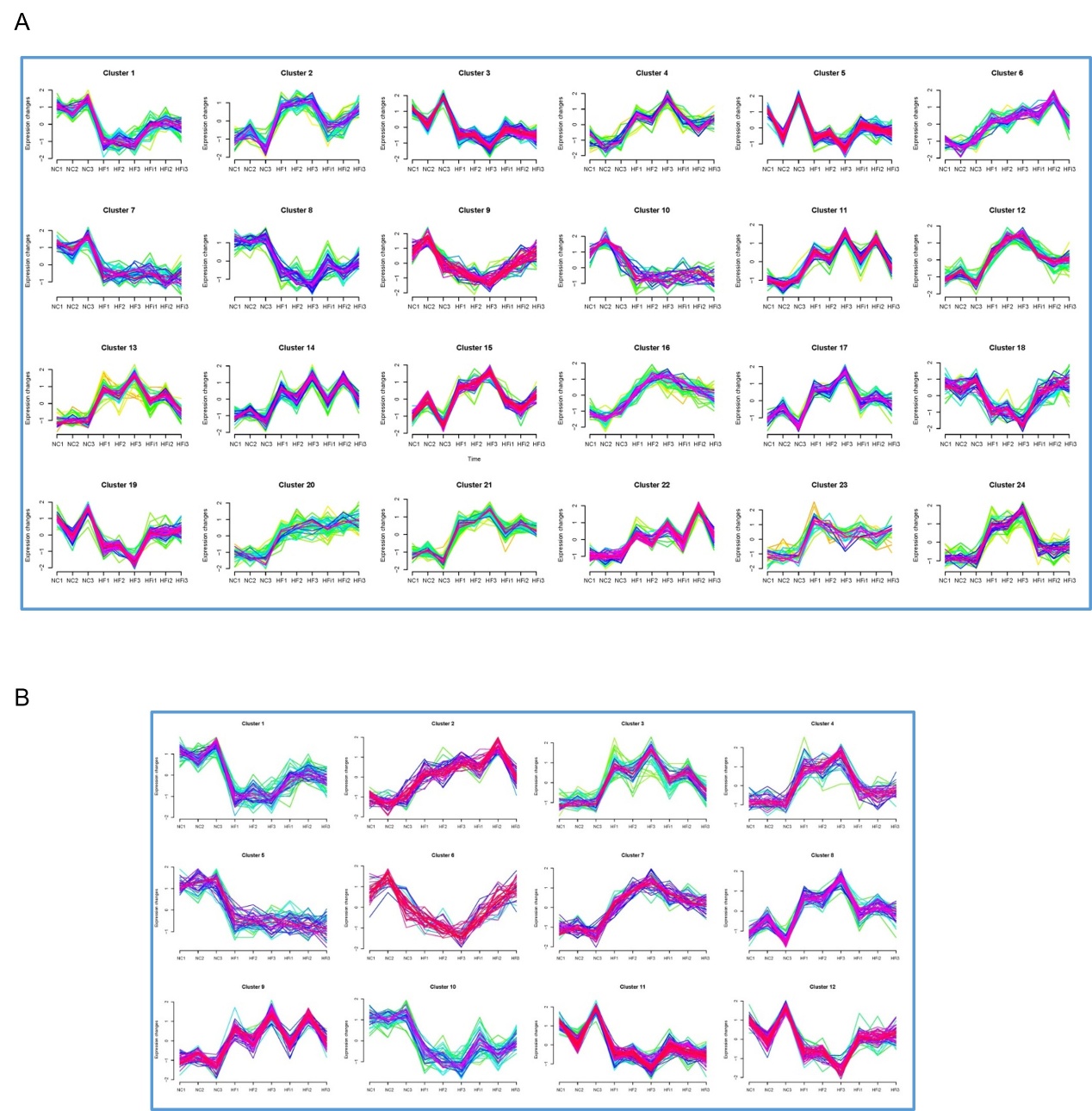


**Figure S3.** **Cluster analysis of gene clusters**. (A and B) Gene co-expression analysis screens out gene clusters with similar trends.
